# Neoadjuvant chemotherapy-induced hemoglobin decline as a prognostic factor in osteosarcoma around the knee joint: a single-center retrospective analysis of 242 patients

**DOI:** 10.1007/s00520-024-08592-2

**Published:** 2024-06-07

**Authors:** Wenxi Yu, Miaoli Sun, Wei Wang, Zan Shen, Yonggang Wang, Hongtao Li

**Affiliations:** 1 Department of Oncology, Shanghai Sixth People’s Hospital Affiliated to Shanghai Jiao Tong University School of Medicine, Shanghai, People’s Republic of China; 2 Department of Clinical Laboratory, Department of Oncology, Shanghai Sixth People’s Hospital Affiliated to Shanghai Jiao Tong University School of Medicine, Shanghai, People’s Republic of China; 3 Department of General Medicine, Department of Oncology, Shanghai Sixth People’s Hospital Affiliated to Shanghai Jiao Tong University School of Medicine, Shanghai, People’s Republic of China; 4 Department of Clinical Laboratory, Jining No.1 People’s Hospital, Jining, Shandong Province People’s Republic of China; 5 Department of Surgery, Affiliated Hospital of Chongqing Population and Family Planning Research Institute, Chongqing, People’s Republic of China

**Keywords:** Osteosarcoma, Anemia, Survival, Tumor necrosis rate

## Abstract

**Purpose:**

Anemia is relatively common in cancer patients, and is associated with poor survival in patients with various malignancies. However, how anemia would affect prognosis and response to neoadjuvant chemotherapy (NAC) in osteosarcoma (OS) is still without substantial evidence.

**Methods:**

We retrospectively analysed 242 patients with stage II OS around the knee joint in our institute. Changed hemoglobin (Hb) levels (before and after NAC) were recorded to assess the prognostic value in DFS (disease-free survival) and tumor response to NAC. Univariate and multivariate analyses were conducted to identify prognostic factors related with outcome in OS patients.

**Results:**

The mean Hb level significantly decreased after NAC (134.5 ± 15.3 g/L *vs.* 117.4 ± 16.3 g/L). The percentage of mild (21%), moderate (4.2%) and severe (0%) anemia patients markedly increased after NAC: 41%, 24% and 4.1% respectively. There was higher percentage of ≥ 5% Hb decline in patients with tumor necrosis rate < 90% (141 out of 161), compared with those with tumor necrosis rate ≥ 90% (59 out of 81). Further univariate and survival analysis demonstrated that Hb decline had a significant role in prediction survival in OS patients. Patients with ≥ 5% Hb decline after NAC had an inferior DFS compared with those with < 5% Hb decline.

**Conclusion:**

In osteosarcoma, patients with greater Hb decrease during neoadjuvant treatment were shown to have worse DFS and a poorer response to NAC than those without. Attempts to correct anemia and their effects on outcomes for osteosarcoma patients should be explored in future studies.

## Introduction

Osteosarcoma (OS) is the most common primary bone cancer in children and adolescent, with a yearly incidence of 5.6 cases per million in children under the age of 15 [1]. Although improvements have been made in molecular and preclinical areas, peri-operative chemotherapy combined with surgery is still the backbone of treatment for OS [2–4]. The mainstay agents used for chemotherapy of OS are high-dose methotrexate, cisplatin, doxorubicin and ifosfamide [2], which are high risk drugs with myelosuppressive effects leading to both neutropenia and anemia [5–8].

Accumulated evidences show that anemia has multiple adverse impacts on the outcome of patients with cancers [9]. Low hemoglobin reduces oxygen supply and intensifies tumor hypoxia, which is a common cause for treatment resistance [10–12]. Anemia in cancer patients is commonly caused by cancer disease or treatment, especially chemotherapy [9]. Regarding to chemotherapy induced anemia (CIA), unfavorable influences were emerged along with treatment. A tendency of lower median tumor response was found in breast cancer patients with CIA [13]. Moreover, the 5-year overall survival are lower in OS patients with continuous decrease in haemoglobin (> 7.6 g/L) during chemotherapy [14].

In osteosarcoma, response to neo-adjuvant chemotherapy(NAC) is histologically graded as good if ≥ 90% of tumor necrosis, or as poor if < 90% of tumor necrosis [15]. Tumor necrosis rate has always been vital and consistently documented prognostic factor and predictor for survival [16, 17]. Since lower hemoglobin results in higher risk of chemo-resistance and 74.4% patients are reported to have anemia during NAC [10–12, 14], how much and to what extend would anemia influence on the efficacy of chemotherapy in OS is still unexplored.

In this study, the incidence of anemia at diagnosis and the proportion of NAC induced anemia of OS patients in a single institute was investigated, the effects of anemia plus change of Hb on tumor response and survival of OS patients undergoing NAC were evaluated.

## Materials and methods

### Patients

The medical data of patients referred to the Shanghai Jiao Tong university affiliated Sixth People’s Hospital with newly diagnosed stage II high-grade osteosarcoma around the knee joint between January 2012 and August 2020. Participants were included in this study if they (a) were pathologically diagnosed with osteosarcoma; (b) received NAC and surgery; (c) had detailed medical data and laboratory results, and (d) had follow‐up results. Participants were excluded in the event of (a) acute injury, infection, and operation history in recent 3 months; (b) severe abnormal liver and kidney function; (c) had blood system diseases or other malignant tumors before treatment; (d) had chronic disease that might cause anemia; (e) had mental illness or cognitive dysfunction. Clinical parameters including gender, age at diagnosis, size/location of tumor, stage of tumor and tumor necrosis rate were recorded in a database in a uniform format.The treatment schedule comprised doxorubicin (60–75 mg/m2), cisplatin (100 mg/m2), and methotrexate (10–12 g/m2) with or without ifosfamide (8–10 g/m2). Chemotherapy-induced necrosis was histologically evaluated based on the tumor necrosis rate. The histologic response was rated as good response if ≥ 90% of tumor necrosis was observed and a poor response was recorded in the event of < 90% tumor necrosis [15].

The hemoglobin, neutrophil, lymphocyte, platelet, and albumin counts were obtained 1 to 7 days before the first chemotherapy treatment was administered, and the final blood samples after chemotherapy were collected within 1 to 14 days before surgery, after an overnight fast. The blood chemical analysis was performed using fully-automatic biochemical analyzer (Hitachi 008AS) and hematology analyzer (Sysmex XN 9000) at the Department of Laboratory Science, Shanghai Sixth People’s Hospital. All data of the blood chemical analysis before the first NAC dose and after the last NAC dose were collected. The anaemia severity are graded into four classes as "No (Normal)", "Mild", "Moderate" and "Severe" anemia based on haemoglobin cut-offs ≥ 120 g/L(120 g/L for men), 110–119 g/L, 80–109 g/L and < 80 g/L in female, according to the World Health Organization’s definition of anemia [18].

### Statistical analysis

R language (version 4.3.2) was utilized for statistical analyses and plots. The Wilcoxon matched-pairs signed-rank test was used for data with a non-normal distribution and comparisons between two groups. Survival analysis of patients was conducted by the Kaplan–Meier method. Univariate and multivariate analyses were evaluated by Cox regression models. A two‐tailed p < 0.05 was considered remarkably significant.

## Results

### Patient characteristics

Between August 2011 and April 2020, a total of 242 stage II patients of high-grade OS around the knee joint were included. The majority were male (62.0%) with a median age of 17 years. 155 patients (64%) had tumor site in the femur, while 11 in fibula (4.5%) and 76 in tibia (31.4%). By definition of Enneking stage, 224 patients (92.6%) were stage IIB while the rest (18,7.4%) were stage IIA. 97 patients (40.1%) had a tumour maximum diameter ≥ 8 cm and the other 145 (59.9%) had a maximum diameter smaller than 8 cm. The majority of patients (66.5%) had a necrosis rate of < 90% and was determined as poor response. The detailed baseline characteristics of the included patients are shown in Table 1.

**Table 1:** Characteristics of included patients

Gender	Overall (*N* = 242)
Male	150 (62.0%)
Female	92 (38.0%)
Age (years)	
Mean (SD)	20.3 (11.4)
Median [Min, Max]	17.0 [3.00, 68.0]
Site of primary tumor	
Femur	155 (64.0%)
Fibula	11 (4.5%)
Tibia	76 (31.4%)
Enneking stage	
IIA	18 (7.4%)
IIB	224 (92.6%)
NAC cycles	
Mean (SD)	4.35 (1.35)
Median [Min, Max]	4.00 [1.00, 8.00]
Tumor diameter	
≥ 8 cm	97 (40.1%)
< 8 cm	145 (59.9%)
Response to chemo	
Poor	161 (66.5%)
Good	81 (33.5%)

### The changes of hemoglobin level before and after NAC

A significant decline was found in Hb level before and after neo-adjuvant chemotherapy. The mean Hb level significantly decreased after NAC (134.5 ± 15.3 g/L vs. 117.4 ± 16.3 g/L, *p* < 0.001, Table 2). None of these patients had hemoglobin level lower than 60 g/L or received blood transfusion after NAC (Table 2).

**Table 2:** The changes of hemoglobin level before and after NAC

Hemoglobin	Before	After
Mean (± SD)	134.537 (± 15.359)	117.372 (± 16.319)
Range	90.000—172.000	65.000—156.000
p < 0.001		

According to 2011 WHO guideline on haemoglobin cut-offs to define anaemia severity, there were 62(25.6%) of patients with mild (21.5%) to moderate (4.1%) anemia at diagnosis, while no patient with severe anemia (Table 3). After neo-adjuvant chemotherapy, 99(41%) patients developed mild anemia, 58 (24%) patients became moderate anemia and 10 (4.1%) patients had severe anemia (p < 0.001, Table 3).

**Table 3:** The changes of anemia severity before and after NAC

Severity	Before		After	
	N	%	N	%
Normal	180	74.4	75	30.9
Mild	52	21.5	99	41
Moderate	10	4.1	58	24
Severe	0	0	10	4.1
Total	242	100	242	100
*p* < 0.001				

### The predict value of changing hemoglobin levels to NAC response

In 161 patients with a necrosis rate of < 90% (poor response), the median Hb level before and after NAC were 136 g/l and 117 g/l, respectively. In 81 patients with a necrosis rate of ≥ 90% (good response), the median Hb level before and after NAC were 132 g/l and 117 g/l, respectively. There was significant difference of the percentage of hemoglobin decline between good and poor response groups (10.4% vs. 13.3%, Table 4, *p* = 0.049). To verify the predictive potential of changing hemoglobin to NAC response, the optimal cut-offs value for percentage of hemoglobin decrease (5%) was evaluated by R package survivalROC. After NAC, there were 200 patients with a ≥ 5% decline of Hb and 42 patients with a < 5% decline of Hb. Besides, in poor response group(*n* = 161), there were more patients a ≥ 5% decline of Hb(*n* = 141), compared with that in good response group (59 out of 81), with a significant p-value 0.004 (Table 5). This result indicated that change of Hb after NAC had a significant role in prediction for the chemo-response in OS patients (Table 5).

**Table 4:** The changes of hemoglobin level before and after NAC between response groups

	Poor (*N* = 161)	Good (*N* = 81)	*p* value
Hb level before NAC			0.045
Mean (SD)	135.938 (15.182)	131.753 (15.423)	
Range	91.000—172.000	90.000—168.000	
Hb level after NAC			0.849
Mean (SD)	117.230 (15.137)	117.654 (18.544)	
Range	70.000—155.000	65.000—156.000	
Changes of Hb level			0.025
Mean (SD)	-18.708 (14.234)	-14.099 (16.492)	
Range	-72.000—20.000	-62.000—24.000	
Changes of percentage			0.049
Mean (SD)	-13.364 (10.118)	-10.387 (12.716)	
Range	-42.105—18.349	-48.819—19.512

**Table 5:** Correlation of chemotherapy induced anemia to necrosis rate

Response to NAC	Hb decline ≥ 5%	Hb decline < 5%
Poor	141(88%)	20(12%)
Good	59(73%)	22(27%)
Total	200(83%)	42(17%)
Pearson’s Chi-squared test, *p* = 0.004		

### Relations between decline of Hb after NAC and outcome in OS

Univariate analysis revealed that several factors were significantly associated with patients' DFS. These candidate variables included sex (*p* = 0.024, HR = 0.6, 95% CI: 0.38 to 0.95), Enneking stage (*p* = 0.006, HR = 3.2, 95% CI: 1.18 to 8.83), response to NAC (good or poor, *p* < 0.001, HR = 0.38, 95% CI: 0.23 to 0.62), tumour diameter (≥ or < 8 cm, *p* < 0.001, HR = 2.81, 95% CI: 1.87 to 4.24) and decline of Hb (*p* = 0.032, HR = 0.53, 95% CI: 0.28 to 0.99). Multivariate Cox regression analysis identified the following factors as significantly associated with DFS: Enneking stage (*p* = 0.024, HR = 2.85, 95% CI: 1.01 to 8.04), response to NAC (good or poor, *p* = 0.003, HR = 0.48, 95% CI: 0.28 to 0.79) and tumour diameter (≥ or < 8 cm, *p* < 0.001, HR = 2.8, 95% CI: 1.88 to 4.29, Table 6).

**Table 6:** Univariate and multivariate analyses for disease-free survival

		Univariable			Multivariable		
Characteristic	N	HR	95% CI	p-value	HR	95% CI	p-value
Sex	242			0.024			
Male		—	—				
Female		0.60	0.38, 0.95				
Enneking stage	242			0.006			0.024
IIA		—	—		—	—	
IIB		3.23	1.18, 8.83		2.85	1.01, 8.04	
Response	242			< 0.001			0.003
Poor		—	—		—	—	
Good		0.38	0.23, 0.62		0.48	0.28, 0.79	
Diameter	242			< 0.001			< 0.001
< 8 cm		—	—		—	—	
≥ 8 cm		2.81	1.87, 4.24		2.84	1.88, 4.29	
Percentage of Hb decline	242			0.032			0.055
≥ 5%		—	—		—	—	
< 5%		0.53	0.28, 0.99		0.55	0.29, 1.06	

**Fig. 1:**
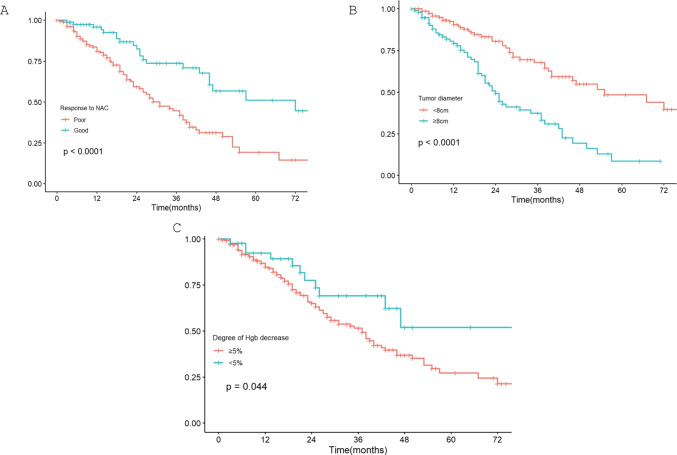
DFS analysis between different factors(**A** Responose to NAC; **B** tumor diameter;**C** Decline of Hb)

Consistent with previous studies, patients with good response to NAC have favorable DFS (median 72 months) than patients with poor response (31 months) (Fig. 1A). According to tumour diameter (Fig. 1B), patients with a tumour diameter ≥ 8 cm have a worse DFS (24 months) than the other patients (55 months).

Subsequently, we analysed the survival outcome by decline of 5% Hb after NAC and demonstrated that patients with ≥ 5% Hb decline have an inferior DFS (mean 44.6 months), compared with those with < 5% Hb decline (63 months, *p* = 0.044, Fig. 1C), which was consistent with our previous finding that patients with severe post-NAC anemia had a worse outcome.

## Discussion

We conducted this retrospective investigation to evaluate the relationship between anemia/decline of Hb and tumor necrosis rate/prognosis. In our cohort of 242 OS patients, 62(25.6%) had anemia at diagnosis while 72 (69.1%) had anemia after NAC. There is a greater likelihood that patients with a fewer decline of Hb would have a better response to NAC, which was further confirmed by survival and univariate analysis.

Anemia is a common diagnosis in patients with cancer that may affect both quality of life and survival. It has several deleterious effects in patients suffered from malignancy. Several studies have reported a negative impact of anemia on disease progression, response to chemo-/radiotherapy, survival, and risk of death [19, 20]. Although anemia may be a consequence of more aggressive forms of cancer, which have worse outcomes, the additional mechanisms unrelated to the tumor may affect prognosis in patients with anemia. Anemia could increase hypoxia in the tumor microenvironment, a condition that is associated with resistance to radiotherapy and chemotherapy, tumor growth, tissue invasion, metastasis and poor outcomes [21]. The anemic state, in proportion to its severity, leads to a set of symptoms, such as dyspnea, fatigue, dizziness, anorexia, lack of concentration, and depressed mood, and these symptoms compromise performance, daily functionality, and quality of life [22–24]. As an additional consequence, adherence to anticancer treatments can be compromised by the reduced performance status due to anemia. Collectively, these factors make anemia a clinically relevant condition in patients with cancer, emphasizing the importance of investigations and management [25].

Several studies have confirmed that Hb concentrations might be a valuable prognostic indicator for prognosis in various cancers [26–29]. In OS, this predictive value of Hb concentrations in survival was still controversial [14]. Recently, alteration of Hb levels during anti-cancer treatment are displayed to be more important prognostic variable than the Hb level at baseline [14, 30, 31]. In our study, relationship between alteration of Hb and prognosis/response to NAC was confirmed by survival and univariate analysis.

Regarding to anemia rectification, blood transfusion, erythropoietin-stimulating agents (ESAs) and iron are three main treatment modalities [32]. Since blood transfusion become more restrictive in mild or moderate anemia after the induction of ESAs, results of clinical trials support the efficacy and safety of ESAs in cancer patients. Despite no sufficient data support the benefits of ESAs in OS patients yet, evidences have shown that administration of ESAs was effective in maintaining hemoglobin levels, increasing response to chemotherapy and improving survival in esophageal cancers. Together with these evidences, results of this study suggest that implementation of ESAs in OS patients with anemia would possibly rectify hemoglobin level, increase response rate and improve prognosis.

It’s reported that over 40% of cancer patients had iron deficiency [33], test to assess iron status is not routinely implemented in this study. It’s rational to extrapolate that more detailed CIA would be profiled if the data of iron status is available. Therefore, future studies on the impact of correcting anemia in patients who suffer from osteosarocma, especially the impact in prognosis and response to NAC, are needed.

The strengths of our study include a cohort who received standard treatments and long follow-up; a focus on the prognostic value in survival and response to chemotherapy of dynamic changes in Hb levels during treatments, and a probing of anemia’s predictive role in survival plus response to chemotherapy. Our study also had some limitations. Firstly, it was a retrospective study, including data from only one institution. Secondly, the number of patients included was not substantial enough to draw concrete conclusions regarding differences.

## Conclusion

In OS, patients with greater Hb decrease during neoadjuvant treatment were shown to have worse DFS and a poorer response to NAC than those without. The role of anemia and the effects of attempts to correct it, in outcomes for osteosarcoma patients should be investigated in future trials.

## Data Availability

The datasets generated during and/or analysed during the current study are available from the corresponding author on reasonable request.
